# Breaking down discipline barriers: an interview with Maël Lebreton on starting a career in an interdisciplinary field

**DOI:** 10.1038/s42003-021-02353-1

**Published:** 2021-07-12

## Abstract

Dr Maël Lebreton is about to set up his own lab at the Paris School of Economics, in September 2021, thanks to an ERC Starting Grant. In the meantime he holds a part-time position at the Swiss Center for Affective Science at the University of Geneva in Switzerland, where he has been a Senior Research Associate since 2018. Mael originally obtained a B.Sc and MSc in Biosciences from the Ecole Normale Superieure de Lyon, and a PhD in Cognitive Neurosciences from Université Pierre et Marie Curie Paris 6 (now Sorbonne Université) in 2013. He then moved to the University of Amsterdam, where he spent over 4 years as a postdoc at the Center for Research in Experimental Economics and Political Decision-Making at the Faculty of Economics and Business. This is where he truly began his independent research career.

**Figure:**
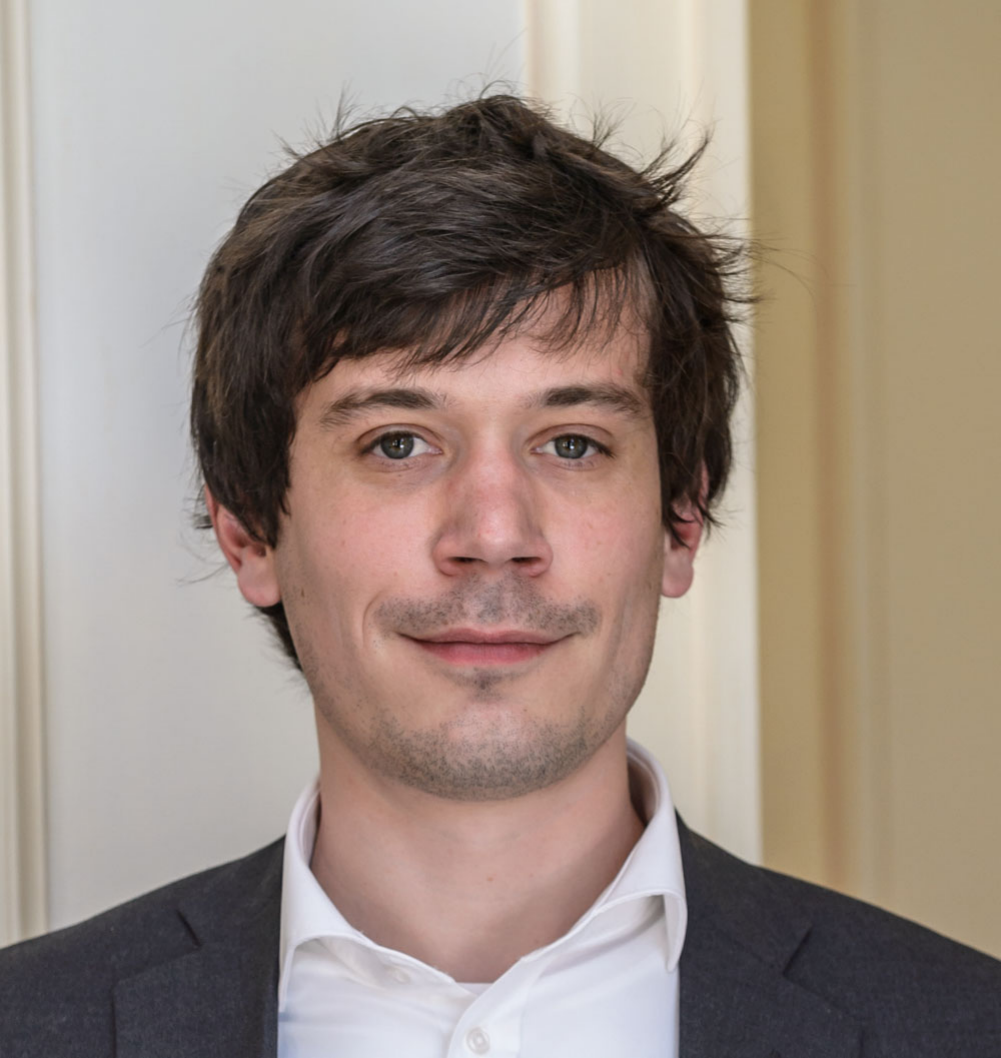
Maël Lebreton

(1) Please tell us about your research interests!

My research focuses on human economic decision making and learning. It lies at the intersection of cognitive neurosciences, behavioral economics, and reinforcement learning. Basically, I am interested in understanding how we decide what to do and how we learn to build a model of the world to support our decision-making. A part of my research aims to isolate some of the cognitive processes at stake in these operations, and I design behavioral experiments to document how these processes shape our behavior in specific situations. A second aspect of my research is focused on proposing computational (i.e., mathematical) models that describe how those processes account for our decisions. We then usually compare those descriptive models to optimal (normative) models of learning and decision-making, to identify where and when—and even sometimes why—our choices are irrational and suboptimal. The final aspect of my research is to investigate the neural correlates of the learning and decision-making processes with human functional neuroimaging.

(2) Neuroeconomics is an exceptionally inter-disciplinary field. What is your advice on establishing suitable collaborations?

In my case, the key was to be patient. It is very natural to underestimate how long it actually takes to understand how to communicate with researchers who have different disciplinary backgrounds (with sometimes a different vocabulary), to identify specific research questions of common interest across disciplinary borders, and to actually find the colleagues that are interested in integrating some new research topics or new methodologies that can be exotic to their usual disciplines. I am always puzzled by the cultural differences that exist between disciplinary fields that nonetheless investigate similar questions—e.g., behavioral economics and cognitive psychology. It seems very difficult for one to overcome those cultural differences and successfully address an audience in a different field without the help and guidance of colleagues well versed in the targeted discipline… but I would recommend it, because interdisciplinary collaborations end up being extremely fun and stimulating.

(3) Any tips on how to make neuroeconomics more approachable for students or a broader audience?

An extremely efficient way to cross interdisciplinary borders is to use (even very simple) formal, mathematical models. In neuroeconomics, such formal models really tie together neuroscience, psychology, economics and cognitive science, around key notions of optimality, efficiency, rationality, etc. This formal, quantitative aspect was really missing in my Biology training curriculum. Not only did this really frustrate me at the time, but I also think it is a strategic mistake—developing an analytical, model-oriented mindset, together with getting the (mathematical) tools to communicate with virtually all other scientific fields (from physics to economics and social sciences) feel, scientifically, priceless.

Regarding the broader audience, our challenge is to communicate complex findings, without intimidating the audience with unnecessarily complex mathematical formulation or neuroscientific jargon. I am a firm believer in the power of data visualization for communicating complex ideas, and I always encourage my students to think of the best way to graphically represent their findings.

Finally, neuroeconomics can also become more approachable by continuing to diversify its focus—e.g., targeting cognitive processes and decision situations that are (more) relevant to our everyday lives, and that address societal issues. In addition, it could be more accessible by working on being more diverse, inclusive, and representative of our society—from the individuals taking part in our studies, to the students and researchers involved in the neuroeconomics community.

(4) Where do you see neuroeconomics going in the next 10 years?

In a way, neuroeconomics has still not delivered on its original ambition and promises: leveraging methods from neuroscience to improve economic models of decision-making. There is a sort of frustration in behavioral economics, to keep documenting judgment and decision biases that describes human behavior but lacks the normative/principled aspects of older models, ultimately constituting a sort of unstructured collection/zoo of behavioral anomalies. In the next 10 years, I feel that research in neuroeconomics will continue its quest toward neurobiological and neurocomputational reductionism: the idea is to understand some core principles of neural coding and constraint as applied to the decision-making process, in order to build mechanistic models that account for the expected, normative aspects of human behavior but also explain away a variety of what seems nowadays like behavioral anomalies.

Another direction that I see neuroeconomics going in over the next 10 years is toward social sciences—with a focus on decision situations, cognitive processes, as well as individuals that are more representative of today’s society and the challenges it faces.

(5) Can you tell us about the challenges you had to face as an ECR who is about to set up a lab during the current pandemic?

First, we are very fortunate to work almost as a “dry” lab: neuroimaging and behavioral data collection usually only happens for a couple of weeks during the year, at dedicated platforms that are independently maintained, and we now even have the opportunity to run behavioral experiments online. Actually, a dominant fraction of our (current) work is actually computational developments and analyses, leveraging already acquired data. So, compared to our colleagues who have to run and maintain “wet” lab with animals/biological samples, the current pandemic has not really hindered our capacity to do most of our research.

The most difficult aspect has been to learn to provide effective supervision to my MSc and PhD students, in the absence of real face-to-face interactions. It is close to impossible to single handedly compensate for the fact that they have been lacking critical aspects of the lab life—i.e., evolving in a rich, stimulating environment, with access to various scientific meetings, the potential for serendipitous collaborations/discussions and day-to-day casual supervision/support. Also, it has been a primary concern of mine to acknowledge that they have been going through difficulties personally, and to provide support and adjust expectations accordingly.

(6) What advice would you give to those wishing to establish an independent career?

First, I would also generally encourage readers of those typical advice sections written by “successful” individuals to be aware of the survivorship bias—nicely illustrated in the xkcd comics: https://xkcd.com/1827/—and read the advice with a critical eye. In this respect, I know that the independent position I enjoy now was (partly) bought with the high-impact papers that I am lucky to have published during my PhD. I have actually been very lucky through my whole career: First, I did my PhD with a really fantastic supervisor (Dr. Mathias Pessiglione), who embarked me on—and entrusted me with—a multitude of exciting and valuable research projects, and who really challenged me intellectually. I was also very lucky to start my career in neuro-economics at a time where this kind of research was really trendy, therefore publishable in very respected and fashionable scientific journals. Finally, I was lucky to secure my first grant pretty early (thanks to the Talent Grant scheme of the Amsterdam Brain Cognition initiative), which initiated the infamous “Matthew effect” in my case—and probably facilitated the obtaining of the next ones.

In addition to this rather sobering view of the academic ecosystem focused on luck, I also have a couple of more positive pieces of advice. The first one is to invest time, effort and energy in finding the right people. A scientifically excellent and supportive supervisor, fun and challenging collaborators, and driven and curious students are all key determinants of success in academics. The collaborative aspect has been, by far, the most rewarding aspect of my career: I am part of multiple collaborations, with a network of early career researchers, which all have been extremely fun, rewarding, and henceforth long standing—sometimes even dating back to my first research internship as a Bachelor student. Close collaborators really provide incredible support to manage the inevitable ups and downs of academic careers, and the scientific values of constantly exchanging or confronting ideas with close peers is inestimable.

The second piece of advice would be to not compromise with scientific quality. In this respect I feel like the young generation is extremely active in embracing open research practices, and other quality-improving actions, which is objectively quite remarkable given the current state of the academic job-market.

And my final piece of advice would be, of course, to have fun. I really feel like the best research comes out of genuine curiosity and unbridled enthusiasm.

*This interview was conducted by Associate Editor Karli Montague-Cardoso and Editorial Board Member Stefano Palminteri*

